# Resource utilization and multidisciplinary care needs for patients with Ehlers–Danlos syndrome

**DOI:** 10.1002/mgg3.2057

**Published:** 2022-09-24

**Authors:** Jordan T. Jones, William R. Black, Wendy Cogan, Elisabeth Callen

**Affiliations:** ^1^ Department of Pediatrics Children's Mercy Kansas City Kansas City Missouri USA; ^2^ Department of Pediatrics University of Missouri‐Kansas City School of Medicine Kansas City Missouri USA; ^3^ Department of Pediatrics University of Kansas School of Medicine Kansas City Kansas USA; ^4^ Center for Children's Healthy Lifestyles and Nutrition Children's Mercy Kansas City Kansas City Missouri USA; ^5^ EDSKC Collaboration Inc, A Non‐Profit Patient Advocacy Organization Kansas City Missouri USA; ^6^ American Academy of Family Physicians Leawood Kansas USA

**Keywords:** adults, Ehlers–Danlos syndrome, health care utilization, multidisciplinary care, pediatrics

## Abstract

**Background:**

Ehlers–Danlos syndrome (EDS) represents a family of heritable connective tissue disorders with overlapping phenotypic features, frequently including joint hypermobility, tissue fragility, and skin hyperextensibility. Comorbid symptoms are common for patients with EDS and include multiple body systems marked by neurologic, cardiovascular, gastrointestinal, musculoskeletal issues, chronic pain, headaches, and anxiety and depression. The many comorbidities lead to high disease burden, which requires greater healthcare utilization.

**Methods:**

This survey of families examines healthcare utilization, of adults and minors, through evaluation of subspecialty care appointments across many healthcare systems in one region.

**Results:**

There were 155 adults and 83 minors with a diagnosis of EDS with a total of 693 unique visits across 27 different specialties at over 20 different hospitals or clinics in the surveyed area. Cardiology, neurology, and gastroenterology were the most utilized subspecialties for adults, while rheumatology, cardiology, and neurology were most utilized by minors. Many respondents (67%) reported their medical care needs are not being met, and 87% reported interest in a multidisciplinary clinic for EDS with the most interest in pain management, physical and occupational therapy, and rheumatology.

**Conclusion:**

Understanding healthcare utilization and needs of those with EDS can provide the foundation for improved care for those with EDS through a coordinated multidisciplinary care model.

## Graphical abstract

Those with Ehlers–Danlos syndrome (EDS) have many comorbid symptoms across multiple body systems. We surveyed patients with EDS and found they are high utilizers of healthcare, but their medical needs are not being met and they are interested in multidisciplinary care to meet their healthcare needs.

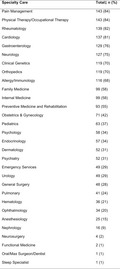

## 1 INTRODUCTION

Ehlers–Danlos Syndrome (EDS) represents a family of heritable connective tissue disorders with overlapping phenotypic features, frequently including joint hypermobility, tissue fragility, and skin hyperextensibility (Bloom et al., 2017; Malfait et al., 2017). Approximately 1 in 2500 to 5000 babies are born with EDS worldwide annually (Joseph et al., 2018). There are 13 recognized distinct clinical subtypes (Malfait et al., 2017), each defined by both major and minor criteria, and there are continued efforts to identify additional genetic subtypes. Definitive diagnosis relies on molecular confirmation with all subtypes except for hypermobile EDS (hEDS), which is a clinical diagnosis (Malfait et al., 2017), though a genetic basis is also suspected for hEDS (Hakim et al., 2004). Comorbid symptoms are common for patients with EDS and include multiple body systems marked by neurologic (Henderson Sr. et al., 2017; Tinkle et al., 2017), cardiovascular (Hakim, O'Callaghan, et al., 2017), gastrointestinal (Fikree et al., 2017), dermatologic (Bowen et al., 2017), gynecologic (Tinkle et al., 2017), and musculoskeletal issues (Castori et al., 2017). Other co‐morbid symptoms, such as chronic pain (Engelbert et al., 2017), deficits with proprioception (Rombaut et al., 2010), headaches (Martin & Neilson, 2014), anxiety and depression (Martin & Neilson, 2014) commonly occur in hEDS. Additionally, some patients are hypermobile and have clinical features consistent with hEDS, but do not fulfill the criteria for hEDS based on the 2017 criteria (Malfait et al., 2017) and are subsequently diagnosed with hypermobility spectrum disorder (HSD).

Due to the complex and comorbid involvement, diagnosis of EDS may be delayed or easily missed, and there is evidence to show that many are misdiagnosed (Hakim, De Wandele, et al., 2017). Diagnostically, differentiating the sub‐groups of EDS can be complex, particularly for those with hEDS and HSD, as these patients have similar presentation and are affected with many similar comorbidities, to such an extent that some have suggested they be considered the same entity with varied clinical severity levels (Aubry‐Rozier et al., 2021). Regardless of whether the diagnosis is hEDS or HSD, the standard treatment involves education and preventative care for symptoms that arise and involves many of the same medical subspecialties (Engelbert et al., 2017). Due to the varied clinical phenotypes in EDS, those with EDS demonstrated increased health care utilization (Williams et al., 2022). To address the many comorbidities and high disease burden in those with EDS, multidisciplinary care has been proposed to help facilitate coordination of care, maximize treatment, and reduce medical visits (Kalisch et al., 2020).

While multidisciplinary care is needed for optimal outcomes in the treatment of those with EDS, it is unclear which specialties are diagnosing EDS, being utilized by those with EDS and what those with EDS feel they need as part of a multidisciplinary care model that can address their needs. The objective of this study was to (1) characterize what specialties diagnosis patients with EDS, (2) determine healthcare utilization by adults and minors with EDS, (3) determine multidisciplinary care interest for those with EDS, and (4) identify specific needs for multidisciplinary care.

## 2 MATERIALS AND METHODS

Using Qualtrics (Provo, Utah) tools hosted at the American Academy of Family Physicians, an electronic survey was sent via an anonymous link to adults with an EDS diagnosis and parents of minors with an EDS diagnosis between March and June 2019. Participants were solicited through EDS support groups within 200 miles of a Mid‐Western metropolitan area. The survey was posted monthly to the support group pages and participants were asked to respond according to their personal experience. The survey contained questions pertaining to patient‐reported EDS subtype, number of minors with EDS in the household, specialty that diagnosed their EDS, specialty care seen previously, medical care needs, interest in multidisciplinary health care for EDS, and how far respondents would be willing to travel to obtain multidisciplinary care for themselves. Survey questions used branching logic, gave multiple choices, while many questions had an “other (please specify)” category for the respondent to fill in responses that may not be listed.

### 2.1 Statistical analyses

The data were analyzed by summarizing binary and categorical variables as frequencies and percentages. All statistical analysis was completed using SPSS 25 (Armonk, NY).

## 3 RESULTS

### 3.1 Demographics

Of 246 respondents that consented to the survey, there were 4 duplicates, and 36 that did not complete any survey questions, which were removed, and 206 responses were analyzed. Of the 206 adult respondents that reported information about themselves they also reported information about 162 minors for a total of 368 subjects. Of respondents, 46% reported on at least one minor and 27% reported on at least 2 minors in their household with a diagnosis of EDS/HSD. Furthermore, 16% of adults and 29% of minors were under evaluation for EDS/HSD at the time of the survey and did not have a diagnosis. The most common diagnosis was hEDS for adults (63%) and minors (62%) followed by HSD for adults (12%) and minors (6%) (Table 1). Most of the adults and minors were diagnosed by genetics (46% for adults and 46% for minors) followed by rheumatology (22% for adults and 24% for minors) (Table 2).

**TABLE 1:** Respondent and minor EDS diagnosis status

Characteristics	Adult *n* (%)	Minor *n* (%)	Total *n* (%)
Classical EDS	12 (7)	4 (4)	16 (4)
Classical‐like EDS	5 (3)	0 (0)	5 (1)
Cardiac‐valvular EDS	1 (1)	0 (0)	1 (<1)
Vascular EDS	5 (3)	1 (1)	6 (2)
Hypermobile EDS	108 (63)	71 (62)	179 (49)
Arthrochalasia EDS	1 (1)	0 (0)	1 (<1)
Kyphoscoliotic EDS	1 (1)	0 (0)	1 (<1)
Spondylodysplastic EDS	1 (1)	0 (0)	1 (<1)
Hypermobility spectrum disorder (HSD)	21 (12)	7 (6)	28 (8)
Currently being evaluated for EDS	29 (14)	41 (25)	70 (19)
Currently being evaluated for HSD	5 (2)	7 (4)	12 (3)
Other	17 (10)	31 (16)	48 (13)

Abbreviation: EDS, Ehlers–Danlos syndrome.

**TABLE 2:** Specialty that diagnosed EDS for respondents and minors

Characteristics	Adult *n* (%)	Minor *n* (%)	Total *n* (%)
Geneticist	86 (46)	52 (46)	138 (46)
Rheumatologist	41 (22)	27 (24)	68 (23)
Physical therapist	4 (2)	0 (0)	4 (1)
Primary care^a^	18 (10)	21 (19)	39 (13)
Orthopedic specialist	3 (2)	1 (1)	4 (1)
Other	37 (20)	12 (11)	49 (16)

Abbreviation: EDS, Ehlers–Danlos syndrome.

^a^ Family Medicine, Internal Medicine, or Pediatrics.

### 3.2 Medical specialty utilization

Almost half (48%) of adults and minors attend one hospital or clinic for their EDS care, while 23% attend two, 10% attend three, 8% attend four and 11% attend five or more different hospitals or clinics for their EDS care. Of the respondents with a diagnosis of EDS/HSD (155 adults and 83 minors), there were a total of 693 unique visits across 27 different specialties at over 20 different hospitals or clinics in the surveyed area. For the 155 adults, there were 584 visits with the most common being cardiology (12%), neurology (8%), gastroenterology (7%), and emergency medicine (7%), while the 83 minors had 109 total visits with the most common being rheumatology (13%), cardiology (11%), neurology (11%), and genetics (11%) (Table 3). There were 92 visits (13%) that took place outside the surveyed region with 85 of those in the adults and 7 in the minors. Of those visits, most were for neurology (11%) and physical and occupational therapy (10%).

**TABLE 3:** Specialty care visits for respondents and minors with EDS

Specialty care	Adult *n* (%)	Minor *n* (%)	Total *n* (%)
Family medicine	36 (6)	2 (2)	38 (5)
Internal medicine	26 (4)	0 (0)	26 (4)
Pediatrics	4 (1)	7 (6)	11 (2)
Clinical genetics	34 (6)	12 (11)	46 (7)
Preventive medicine and rehabilitation	7 (1)	4 (4)	11 (2)
Physical therapy/occupational therapy	27 (5)	9 (8)	36 (5)
Orthopedics	33 (6)	8 (7)	41 (6)
Neurology	47 (8)	12 (11)	59 (9)
Cardiology	72 (12)	12 (11)	84 (12)
Gastroenterology	41 (7)	5 (5)	46 (7)
General surgery	25 (4)	0 (0)	25 (4)
Pain management	26 (4)	4 (4)	30 (4)
Allergy/immunology	13 (2)	1 (1)	14 (2)
Rheumatology	22 (4)	14 (13)	36 (5)
Hematology	8 (1)	1 (1)	9 (1)
Dermatology	6 (1)	0 (0)	6 (1)
Obstetrics & gynecology	29 (5)	1 (1)	30 (4)
Urology	8 (1)	3 (3)	11 (2)
Psychology	5 (1)	2 (2)	7 (1)
Psychiatry	8 (1)	1 (1)	9 (1)
emergency services	41 (7)	5 (5)	46 (7)
Anesthesiology	12 (2)	1 (1)	13 (2)
Endocrinology	14 (2)	0 (0)	14 (2)
Nephrology	4 (1)	1 (1)	5 (1)
Ophthalmology	10 (2)	2 (2)	12 (2)
Pulmonary	10 (2)	1 (1)	11 (2)
Other	16 (3)	1 (1)	17 (2)
Total visits	584	109	693

Abbreviation: EDS, Ehlers–Danlos syndrome.

### 3.3 Medical specialty needs

When the respondents were asked if their current EDS/HSD medical care needs were being met, 67% responded “No,” 23% responded “Maybe” and 10% responded “Yes,” while 87% responded that it would be beneficial for them to receive care from a multidisciplinary clinic for EDS/HSD. Respondents were asked which specialties they would include in a hypothetical multidisciplinary clinic for EDS/HSD and 84% responded pain management, 84% responded physical and occupational therapy, 82% responded rheumatology, 81% responded cardiology, 76% responded gastroenterology, and 75% responded neurology (Table 4). Additionally, respondents were asked how far they would be willing to travel to obtain care at an EDS/HSD multidisciplinary clinic and 8% responded 15 minutes or less, 34% responded 30–60 minutes, 22% responded 60–120 minutes, 22% responded more than 120 minutes and 15% responded they would be willing to fly to obtain care.

**TABLE 4:** Specialties those with EDS would prefer in a multidisciplinary clinic

Specialty care	Total^a^ *n* (%)
Pain management	143 (84)
Physical therapy/occupational therapy	143 (84)
Rheumatology	139 (82)
Cardiology	137 (81)
Gastroenterology	129 (76)
Neurology	127 (75)
Clinical genetics	119 (70)
Orthopedics	119 (70)
Allergy/immunology	116 (68)
Family medicine	99 (58)
Internal medicine	99 (58)
Preventive medicine and rehabilitation	93 (55)
Obstetrics & gynecology	71 (42)
Pediatrics	63 (37)
Psychology	58 (34)
Endocrinology	57 (34)
Dermatology	52 (31)
Psychiatry	52 (31)
Emergency services	49 (29)
Urology	49 (29)
General surgery	48 (28)
Pulmonary	41 (24)
Hematology	36 (21)
Ophthalmology	34 (20)
Anesthesiology	25 (15)
Nephrology	16 (9)
Neurosurgery	4 (2)
Functional medicine	2 (1)
Oral/max surgeon/dentist	1 (1)
Sleep specialist	1 (1)

Abbreviation: EDS, Ehlers–Danlos syndrome.

^a^ Total number of respondents *n* = 178.

## 4 DISCUSSION

Ehlers–Danlos syndrome (EDS) and hypermobility spectrum disorder (HSD) have become more widely recognized along with the increased disease burden that occurs from multiple comorbidities commonly present in those with EDS/HSD. This results in referrals to several specialty care services, which increased health care utilization and lacks integration between treatment plans. Multidisciplinary care can help facilitate care coordination, streamline medical visits, and improve health outcomes, however, there are few standards of care for those with EDS/HSD, which results in disjointed care, particularly when individual specialty disciplines are sought without a mechanism for integrating care. Unfortunately, this integration often falls upon the parents and families, further increasing their treatment burden. This study provides information about diagnosis and utilization of care for those with EDS/HSD and highlights the perceived needs of those with EDS/HSD with the goal to inform a multidisciplinary standard of care model.

Almost all EDS subtypes were represented in this study, however, hypermobile EDS (hEDS) and HSD were the most common, which is consistent with the current literature (Tinkle et al., 2017). This is particularly noteworthy, as HSD was a relatively new diagnosis during the timeframe in which this study was conducted. Those with EDS in this specific Mid‐Western region are mostly diagnosed by geneticists, however, many are also diagnosed by rheumatologists and other specialists. This would suggest that other specialties are capable of diagnosing EDS/HSD in the absence of geneticists. Additionally, there is a nationwide shortage of geneticists (Jenkins et al., 2021), and in the absence of diagnosis and referral for appropriate care, symptoms of fatigue worsen, and physical deconditioning and mental health can deteriorate quality of life (QoL), increasing the need for more aggressive and costly rehabilitation therapy (Hakim, De Wandele, et al., 2017). Education about EDS/HSD diagnosis and care provided to commonly utilized specialties may provide earlier diagnosis and appropriate referrals for those with EDS/HSD.

The survey respondents with EDS/HSD had a high utilization of clinical care with multiple visits to specialty care across many subspecialties and health care systems. Adults utilized more subspecialty care from cardiology, neurology, and gastroenterology, which represents specific needs for those with EDS/HSD. This likely represents common comorbidities that require care (Tinkle et al., 2017), but also may represent specialty utilization that is easier to obtain due to the offerings of the local healthcare systems. Minors also utilized a lot of subspecialty care that included rheumatology, cardiology, neurology, and genetics. This likely represents more parental focus on evaluation and diagnosis of EDS in minors as there were more minors undergoing evaluation for EDS and HSD in this study. A previous study showed that health care utilization for those with EDS/HSD shifted from more diagnostic needs to more management of disease over time, however, the utilization of care remained high (Williams et al., 2022). Additionally, the more commonly utilized subspecialty care may represent a place to implement education about EDS/HSD to improve care and outcomes, but also may be ideal specialties to target for multidisciplinary care.

In this study, there was a high burden of disease that was quantified by respondents' utilization of care for themselves and minors. Many respondents reported pursuing simultaneous care at three or more different hospitals and expressed willingness to travel more than an hour away for their EDS care. While psychological burden was not specifically assessed in this study, this degree of health care utilization is likely a psychological burden for adults as this is a heritable condition and there are multiple family members in the home that are affected. Respondents overwhelmingly noted that their needs are not being met for themselves and their children, which may be due to the experienced burden of self‐care, self‐advocacy, and care for other family members in the home with EDS/HSD, particularly in context of non‐integrated care. There are many psychological conditions associated with EDS/HSD (Bulbena et al., 2017) and this could be, in part, due to the psychological burden of caring for oneself and others with EDS/HSD. Further research is needed to assess the degree of psychological burden in adults and minors with EDS/HSD.

Multidisciplinary care is clearly the goal for chronic, complex medical conditions, and most respondents are interested in a multidisciplinary clinic for care and management of EDS/HSD. The top five requested specialties for a multidisciplinary clinic from patients with EDS/HSD are pain management, physical and occupational therapy (PT/OT), rheumatology, cardiology, and gastroenterology, but there were several other highly requested specialties too (Table 4). Interestingly, cardiology and gastroenterology are already specialties that are commonly utilized, which likely indicates that these are highly needed areas for those with EDS/HSD or that respondents would like to consolidate their current specialty care into a multidisciplinary clinic. Rheumatology was a specialty more commonly utilized by minors compared to adults in this study, which may represent easier access to pediatric rheumatology versus adult rheumatology or pediatric rheumatology that is more aware and engaged in the care for EDS/HSD in the surveyed region. Chronic musculoskeletal pain is one of the most common complaints in those with EDS (Malfait et al., 2021), which is likely why pain management, PT/OT, and rheumatology are the top three requested specialties as they all share a common focus on musculoskeletal health. Almost 60% of respondents felt primary care (family medicine, internal medicine, pediatrics) would be important in a multidisciplinary team; however, primary care was poorly utilized for care by those with EDS/HSD and the emergency department had higher utilization than primary care based on responses. This paradigm may arise from poor awareness, insufficient knowledge, or discomfort from primary care clinicians in managing those with EDS/HSD (Jones & Black, 2022). Alternatively, while PCPs are recognized as an important part of their EDS care coordination, the initial ability of PCPs to meet their needs may be limited by the amount of time available for a single patient visit and/or the increased frequency of visits needed to care for the multiple comorbid symptoms common with EDS/HSD.

While multidisciplinary care may be the goal to facilitate coordination of care, maximize treatment, and reduce medical visits for those with complex disease, it faces many challenges, which include coordination of multiple services, interest and knowledge of specific conditions, and financial burdens. Respondents report willingness to travel to obtain multidisciplinary care that will address their needs, but the services may not exist. The willingness to travel does demonstrate the current need, gaps in care, and lack of standard of care for those with EDS/HSD. This study also gives a framework for a multidisciplinary clinic model that is endorsed by those with EDS/HSD. Interestingly, this question focused on the parent and their willingness to travel for their care but did not take into consideration a parent's willingness to travel the same time frame or different time frame for their minors who require care. As such, it may be a reasonable expectation that parents would be willing to go to greater lengths to pursue care for their children than for themselves.

Our study has several limitations, which includes that the survey was completed regionally, and the findings may not be generalizable to larger groups of adults and minors with EDS/HSD in different geographical regions. Additionally, regional resources may be different, which may change the health care utilization of those with EDS/HSD, however, the utilization reported here does include subspecialty care that is associated with increased comorbidities commonly reported in those with EDS/HSD and would likely be the same regardless of region of the country. Exact number of visits with each specialist was not collected, and unique visits reported only represents one visit per adult or minor, so the exact number of visits likely is higher than reported here. Recall bias is also possible, and responses may be recalled inaccurately. The study area is sizable and has numerous health care systems and subspecialty care and smaller areas with fewer resources may provide similar care from less specialties or be more reliant on primary care clinicians. Additionally, while families expressed that their current needs were not being met, this study was not able to follow‐up and better delineate why their needs were unmet, requiring some informed speculation pertaining to patient‐experienced treatment burden.

## 5 CONCLUSION

Findings from this study suggest that there are several specialties that diagnose EDS/HSD, and similar to previous reports, health care utilization among adults and minors with EDS/HSD is high due to the many comorbidities that occur. Many respondents obtain care from several subspecialties across many medical institutions which may lead to fractured, poorly coordinated care that does not meet the patient's needs, and leads to lack of trust in healthcare providers and negative expectations for healthcare needs (Langhinrichsen‐Rohling et al., 2021). This represents a unique opportunity to develop specialized multidisciplinary care for adults and minors with EDS/HSD, that can address their specific needs and provide a foundation to better understand the patients' needs and develop better, coordinated care to meet the needs of those with EDS/HSD.

## AUTHOR CONTRIBUTIONS

*Conceptualization of study*: Jordan T. Jones, William R. Black, Wendy Cogan, Elisabeth Callen *Data acquisition*: Wendy Cogan, Elisabeth Callen, *Data analysis and Manuscript preparation*: Jordan T. Jones, William R. Black, Wendy Cogan, Elisabeth Callen.

## FUNDING INFORMATION

None.

## CONFLICT OF INTEREST

The authors have no conflicts of interest to disclose. No honorarium, grant, or payment was given to any author to produce this work.

## ETHICS STATEMENT

This work was conducted in accordance with the Declaration of Helsinki. Institutional review board approval was obtained from American Academy of Family Physicians (IRB Application # 19‐335). All respondents were 18 years old or older and consented when they voluntarily completed the survey.

## Data Availability

The data that support the findings of this study are available from the corresponding author upon reasonable request.

## References

[mgg32057-bib-0001] Aubry‐Rozier, B. , Schwitzguebel, A. , Valerio, F. , Tanniger, J. , Paquier, C. , Berna, C. , Hügle, T. , & Benaim, C. (2021). Are patients with hypermobile Ehlers–Danlos syndrome or hypermobility spectrum disorder so different? Rheumatology International, 41(10), 1785–1794. 10.1007/s00296-021-04968-3 34398260 PMC8390400

[mgg32057-bib-0002] Bloom, L. , Byers, P. , Francomano, C. , Tinkle, B. , Malfait, F. , & Steering Committee of The International Consortium on the Ehlers–Danlos Syndromes . (2017). The international consortium on the Ehlers–Danlos syndromes. American Journal of Medical Genetics. Part C, Seminars in Medical Genetics, 175(1), 5–7. 10.1002/ajmg.c.31547 28306227

[mgg32057-bib-0003] Bowen, J. M. , Sobey, G. J. , Burrows, N. P. , Colombi, M. , Lavallee, M. E. , Malfait, F. , & Francomano, C. A. (2017). Ehlers–Danlos syndrome, classical type. American Journal of Medical Genetics. Part C, Seminars in Medical Genetics, 175(1), 27–39. 10.1002/ajmg.c.31548 28192633

[mgg32057-bib-0004] Bulbena, A. , Baeza‐Velasco, C. , Bulbena‐Cabre, A. , Pailhez, G. , Critchley, H. , Chopra, P. , Mallorquí‐Bagué, N. , Frank, C. , & Porges, S. (2017). Psychiatric and psychological aspects in the Ehlers–Danlos syndromes. American Journal of Medical Genetics. Part C, Seminars in Medical Genetics, 175(1), 237–245. 10.1002/ajmg.c.31544 28186381

[mgg32057-bib-0005] Castori, M. , Tinkle, B. , Levy, H. , Grahame, R. , Malfait, F. , & Hakim, A. (2017). A framework for the classification of joint hypermobility and related conditions. American Journal of Medical Genetics. Part C, Seminars in Medical Genetics, 175(1), 148–157. 10.1002/ajmg.c.31539 28145606

[mgg32057-bib-0006] Engelbert, R. H. , Juul‐Kristensen, B. , Pacey, V. , de Wandele, I. , Smeenk, S. , Woinarosky, N. , Sabo, S. , Scheper, M. C. , Russek, L. , & Simmonds, J. V. (2017). The evidence‐based rationale for physical therapy treatment of children, adolescents, and adults diagnosed with joint hypermobility syndrome/hypermobile Ehlers–Danlos syndrome. American Journal of Medical Genetics. Part C, Seminars in Medical Genetics, 175(1), 158–167. 10.1002/ajmg.c.31545 28306230

[mgg32057-bib-0007] Fikree, A. , Chelimsky, G. , Collins, H. , Kovacic, K. , & Aziz, Q. (2017). Gastrointestinal involvement in the Ehlers–Danlos syndromes. American Journal of Medical Genetics. Part C, Seminars in Medical Genetics, 175(1), 181–187. 10.1002/ajmg.c.31546 28186368

[mgg32057-bib-0008] Hakim, A. , De Wandele, I. , O'Callaghan, C. , Pocinki, A. , & Rowe, P. (2017). Chronic fatigue in Ehlers–Danlos syndrome‐hypermobile type. American Journal of Medical Genetics. Part C, Seminars in Medical Genetics, 175(1), 175–180. 10.1002/ajmg.c.31542 28186393

[mgg32057-bib-0009] Hakim, A. , O'Callaghan, C. , De Wandele, I. , Stiles, L. , Pocinki, A. , & Rowe, P. (2017). Cardiovascular autonomic dysfunction in Ehlers–Danlos syndrome‐hypermobile type. American Journal of Medical Genetics. Part C, Seminars in Medical Genetics, 175(1), 168–174. 10.1002/ajmg.c.31543 28160388

[mgg32057-bib-0010] Hakim, A. J. , Cherkas, L. F. , Grahame, R. , Spector, T. D. , & MacGregor, A. J. (2004). The genetic epidemiology of joint hypermobility: A population study of female twins. Arthritis and Rheumatism, 50(8), 2640–2644. 10.1002/art.20376 15334479

[mgg32057-bib-0011] Henderson, F. C., Sr. , Austin, C. , Benzel, E. , Bolognese, P. , Ellenbogen, R. , Francomano, C. A. , Ireton, C. , Klinge, P. , Koby, M. , Long, D. , Patel, S. , Singman, E. L. , & Voermans, N. C. (2017). Neurological and spinal manifestations of the Ehlers–Danlos syndromes. American Journal of Medical Genetics. Part C, Seminars in Medical Genetics, 175(1), 195–211. 10.1002/ajmg.c.31549 28220607

[mgg32057-bib-0012] Jenkins, B. D. , Fischer, C. G. , Polito, C. A. , Maiese, D. R. , Keehn, A. S. , Lyon, M. , Edick, M. J. , Taylor, M. R. G. , Andersson, H. C. , Bodurtha, J. N. , Blitzer, M. G. , Muenke, M. , & Watson, M. S. (2021). The 2019 US medical genetics workforce: A focus on clinical genetics. Genetics in Medicine, 23(8), 1458–1464. 10.1038/s41436-021-01162-5 33941882 PMC8091643

[mgg32057-bib-0013] Jones, J. T. , & Black, W. R. (2022). Provider knowledge and experience in care, management, and education of pediatric Ehlers–Danlos syndrome. Global Pediatric Health, 9, 2333794X221112841. 10.1177/2333794X221112841 PMC9305803 35873707

[mgg32057-bib-0014] Joseph, A. W. , Joseph, S. S. , Francomano, C. A. , & Kontis, T. C. (2018). Characteristics, diagnosis, and Management of Ehlers–Danlos Syndromes: A review. JAMA Facial Plastic Surgery, 20(1), 70–75. 10.1001/jamafacial.2017.0793 29121166

[mgg32057-bib-0015] Kalisch, L. , Hamonet, C. , Bourdon, C. , Montalescot, L. , de Cazotte, C. , & Baeza‐Velasco, C. (2020). Predictors of pain and mobility disability in the hypermobile Ehlers–Danlos syndrome. Disability and Rehabilitation, 42(25), 3679–3686. 10.1080/09638288.2019.1608595 31060411

[mgg32057-bib-0016] Langhinrichsen‐Rohling, J. , Lewis, C. L. , McCabe, S. , Lathan, E. C. , Agnew, G. A. , Selwyn, C. N. , & Gigler, M. E. (2021). They've been BITTEN: Reports of institutional and provider betrayal and links with Ehlers–Danlos syndrome patients' current symptoms, unmet needs and healthcare expectations. Therapeutic Advances in Rare Disease, 2, 26330040211022033. 10.1177/26330040211022033 PMC10032464 37181101

[mgg32057-bib-0017] Malfait, F. , Colman, M. , Vroman, R. , De Wandele, I. , Rombaut, L. , Miller, R. E. , Malfait, A. M. , & Syx, D. (2021). Pain in the Ehlers–Danlos syndromes: Mechanisms, models, and challenges. American Journal of Medical Genetics. Part C, Seminars in Medical Genetics, 187(4), 429–445. 10.1002/ajmg.c.31950 34797601

[mgg32057-bib-0018] Malfait, F. , Francomano, C. , Byers, P. , Belmont, J. , Berglund, B. , Black, J. , Bloom, L. , Bowen, J. M. , Brady, A. F. , Burrows, N. P. , Castori, M. , Cohen, H. , Colombi, M. , Demirdas, S. , De Backer, J. , De Paepe, A. , Fournel‐Gigleux, S. , Frank, M. , Ghali, N. , … Tinkle, B. (2017). The 2017 international classification of the Ehlers–Danlos syndromes. American Journal of Medical Genetics. Part C, Seminars in Medical Genetics, 175(1), 8–26. 10.1002/ajmg.c.31552 28306229

[mgg32057-bib-0019] Martin, V. T. , & Neilson, D. (2014). Joint hypermobility and headache: the glue that binds the two together‐‐part 2. Headache, 54(8), 1403–1411. 10.1111/head.12417 24958300

[mgg32057-bib-0020] Rombaut, L. , De Paepe, A. , Malfait, F. , Cools, A. , & Calders, P. (2010). Joint position sense and vibratory perception sense in patients with Ehlers–Danlos syndrome type III (hypermobility type). Clinical Rheumatology, 29(3), 289–295. 10.1007/s10067-009-1320-y 19937459

[mgg32057-bib-0021] Tinkle, B. , Castori, M. , Berglund, B. , Cohen, H. , Grahame, R. , Kazkaz, H. , & Levy, H. (2017). Hypermobile Ehlers–Danlos syndrome (a.k.a. Ehlers–Danlos syndrome type III and Ehlers–Danlos syndrome hypermobility type): Clinical description and natural history. American Journal of Medical Genetics. Part C, Seminars in Medical Genetics, 175(1), 48–69. 10.1002/ajmg.c.31538 28145611

[mgg32057-bib-0022] Williams, S. E. , Tran, S. T. , Lynch‐Jordan, A. , Goldschneider, K. R. , Ting, T. V. , Kashikar‐Zuck, S. , & Neilson, D. (2022). Healthcare utilization among youth with Ehlers–Danlos syndrome hypermobile type. American Journal of Medical Genetics. Part A, 188(4), 1109–1117. 10.1002/ajmg.a.62625 34989147

